# Total thyroidectomy as a method of choice in the treatment of Graves’ disease - analysis of 1432 patients

**DOI:** 10.1186/s12893-015-0023-3

**Published:** 2015-04-09

**Authors:** Toplica Bojic, Ivan Paunovic, Aleksandar Diklic, Vladan Zivaljevic, Goran Zoric, Nevena Kalezic, Vera Sabljak, Nikola Slijepcevic, Katarina Tausanovic, Nebojsa Djordjevic, Dragana Budjevac, Lidija Djordjevic, Aleksandar Karanikolic

**Affiliations:** Department for Endocrine Surgery and Breast Surgery, Clinic of General Surgery, Clinical Centre Nis, Bul. Dr Zorana Djindjica 48, 18000 Nis, Serbia; University of Nis School of Medicine, Bul. Dr Zorana Djindjica 81, 18000 Nis, Serbia; Centre for Endocrine Surgery, Clinic of Endocrinology Diabetes and Metabolic Disease, Clinical Centre Serbia, Dr Subotica 13, 11000 Belgrade, Serbia; University of Belgrade School of Medicine, Dr Subotica 8, 11000 Belgrade, Serbia; Centre for Anaesthesia and Resuscitation, Clinical Centre Serbia, Pasterova 2, 11000 Belgrade, Serbia

**Keywords:** Graves’ disease, Total thyroidectomy, Carcinoma, Complications

## Abstract

**Background:**

Graves’ disease represents an autoimmune disease of the thyroid gland where surgery has an important role in its treatment. The aim of our paper was to analyze the results of surgical treatment, the frequency of microcarcinoma and carcinoma, as well as to compare surgical complications in relation to the various types of operations performed for Graves’ disease.

**Methods:**

We analysed 1432 patients (221 male and 1211 female) who underwent surgery for Graves’ disease at the Centre for Endocrine Surgery in Belgrade during 15 years (1996–2010). Average age was 34.8 years. Frequency of surgical complications within the groups was analyzed with nonparametric Fisher’s test.

**Results:**

Total thyroidectomy (TT) was performed in 974 (68%) patients, and Dunhill operation (D) in 221 (15.4). Carcinoma of thyroid gland was found in 146 patients (10.2%), of which 129 (9%) were a microcarcinoma. Complication rates were higher in the TT group, where there were 31 (3.2%) patients with permanent hypoparathyroidism, 9 (0.9%) patients with unilateral recurrent nerve paralysis and 10 (1.0%) patients with postoperative bleeding. Combined complications, such as permanent hypoparathyroidism with bleeding were more common in the D group where there were 2 patients (0,9%), while unilateral recurrent nerve paralysis with bleeding was more common in the TT group where there were 3 cases (0,3%).

**Conclusions:**

Frequency of complications were not significantly statistically different in relation to the type of surgical procedure. Total thyroidectomy represents a safe and efficient method for treating patients with Graves’ disease, and it is not followed by a greater frequency of complications in relation to less extensive procedures.

## Background

Graves’ disease (GD) represents an autoimmune disease of the thyroid gland, which is manifested by excessive production of hormones of the thyroid gland and is a common cause of hyperthyroidism. GD is primarily treated by anti thyroid drugs, and only in the case of failure of this method of treatment are other types of therapy, such as the use of radioactive iodine, taken into account [1]. In some countries, such as the United States, total thyroidectomy is recommended only after treatment with radioactive iodine. In Scandinavia, however, surgery with a low complication rate plays an important role in the treatment of GD [2,3].

Surgical treatment is applied after exhausting other treatment modalities, and represents a definitive treatment where the reasons for surgery are various. The rate of relapse after surgery is significantly lower than for treatment with radioiodine, or anti thyroid drugs [4,5]. Antithyroid drugs on one hand have good tolerance, but the rate of recurrence of hyperthyroidism is up to 69% after termination of the treatment [6]. Radio ablation is a cost effective method, good for patients at risk for surgery, but in order to achieve euthyroidism it takes up to 6 month [7].

Surgical interventions, on the other hand, carried out in patients with significant goitre and ophthalmopathy give the fastest results and lead to the rapid regression of the symptoms of hyperthyreosis [8].

Surgical treatment of GD achieves a quick and efficient therapeutic effect and interventions performed by an experienced surgeon are considered a safe procedure [1]. The surgical treatment of GD includes several modalities of surgical operations such as bilateral subtotal lobectomy (STT), unilateral total lobectomy and contralateral subtotal lobectomy - Dunhill’s operation (D) and total or near total thyroidectomy (TT).

The aim of our work was to analyze the results of surgical treatment of patients with GD, the frequency of microcarcinoma and carcinoma in patients who underwent surgery for GD, as well as to compare surgical complications (postoperative bleeding, postoperative hypoparathyroidism, lesions of the recurrent laryngeal nerve) in relation to the various types of operations performed for GD.

## Methods

The research is a retrospective study in which data were analyzed for patients surgically treated for GD at the Centre for Endocrine Surgery, Clinical Centre Serbia in the period 1996–2010. In this period a total of 1432 patients were operated for GD. Among the operated patients there were significantly more women, 1211 (84.6%), while there were 221 men (15.4%); which roughly corresponds to a ratio of 6 to 1. The average age of the patients was 34.8 years (21 to 76 years).

All patients had previously been treated with thyrostatic therapy (carbimazole, thiamazole, propylthiouracil) for a period of 2 to 7 years, as well as with β-adrenergic blockers. There were no patients who were previously treated with radioiodine.

The diagnosis was based on clinical presentation, thyroid hormones (T3, T4, TSH), and values of TSH-receptor antibodies (increase above 1.0 IU/L).

Preoperatively, all patients achieved an euthyroid state and were afterwards reviewed by an anaesthesiologist, cardiologist and otorhinolaryngologist. All patients were treated preoperatively with Lugol’s solution of iodine with 3–5 drops of iodine/a day for 2–3 weeks before surgery.

The study has been performed in accordance with the Declaration of Helsinki. Ethical approval for our study was obtained from institutional ethical committee of Clinical Centre Belgrade, Serbia. All patients signed a consent form for the surgical procedure which comprises their consent for the storage and use of their data.

Data analyzed were patient sex, age, indications for surgery, type of surgery, the incidence of carcinoma and microcarcinoma and the frequency of complications (postoperative permanent hypoparathyroidism, unilateral recurrent nerve paralysis and postoperative hematomas), as well as the occurrence of associated complications with the same patient.

Postoperative permanent hypoparathyroidism was confirmed 3 months after surgery and was defined by the values of the parathyroid hormone below the lower limit of the normal range of hormones (normal parathyroid hormone from 10.0 to 65.0 pg/mL).

Unilateral recurrent nerve paralysis was confirmed by the existence of unilateral vocal cord paralysis.

Postoperative hematoma was diagnosed over a period of 24 hours after surgery and required revision of haemostasis.

The analysis used data from the medical records, operational protocols, reports on the histopathological examinations and check-ups.

Obtained data (incidence of complications and the incidence of associated complications), are presented in tables with absolute numbers and the percentages of corresponding structure in relation to the type of applied surgical methods. Comparison between the frequencies was performed with nonparametric Fisher’s test. Statistical analysis was performed with software package SPSS version 18 (SPSS Inc., Chicago, Illinois, USA) at the level of statistical significance of p < 0.05.

## Results and discussion

Table 1 shows the indications for surgical treatment of GD, which included thyromegaly, confirmed nodule >1 cm in diameter, ophthalmopathy and resistance to antithyroid drug therapy. The indication for surgery in the majority of 831 (65%) patients operated for GD was thyromegaly, while a smaller number, 143 patients (10%), were related to ophthalmopathy and an ultrasound diagnosed nodule >1 cm.

**Table 1:** **Indications for surgical treatment for Graves’ disease**

**Indications**	**No**
Thyromegaly	931 (65%)
Having established nodule > 1 cm	143 (10%)
Graves’ ophthalmopathy	143 (10%)
Anti thyroid resistance to therapy, anti thyroid adverse effects of therapy, the inability of an adequate antithyroid therapy	215 (15%)
Σ	1432 (100%)

Table 2 shows the distribution of patients according to the type of operation. During the studied 15-year period, in 974 patients (68%) the operation of choice for GD was TT, while D was the operation of choice in only 221 (15.4%) patients. STT was performed only in the first half of the studied period. With the development of surgical technique this procedure was abandoned. At our institution, the remnant of tissue which is normally left after STT is approximately 2 g on each side, while after D its approximately 3 g on one side only.

**Table 2:** **Distribution of operated according to the type of operation for Graves’ disease**

**Year**	**96**	**97**	**98**	**99**	**0**	**1**	**2**	**3**	**4**	**5**	**6**	**7**	**8**	**9**	**10**	**Σ**	**%**
TT	5	11	25	33	40	47	55	90	86	97	95	91	88	89	122	974	68
D	20	22	25	25	44	43	32	5	3	0	0	1	1	0	0	221	15.4
STT	65	60	54	30	15	8	5	0	0	0	0	0	0	0	0	237	16.6
Σ	90	93	88	99	98	92	95	89	89	97	95	92	89	89	122	1432	100

Table 3 shows the incidence of thyroid cancer in patients operated for GD. In 145 patients (10.2%) with GD, definitive pathohistological examination revealed thyroid cancer. Carcinomas of less than 1 cm (microcarcinomas) were represented in 129 patients (89.8%), while greater than 1 cm were present in 17 patients (1.2%). In the remaining 1286 patients (89.8%) we did not observe the existence of cancer.

**Table 3:** **The incidence of thyroid cancer in patients operated for Graves’ disease**

**Tumor size**	**N** ^**0**^	**%**
Tumor < 1 cm	129	9.0
Tumor > 1 cm	17	1.2
Without tumor	1286	89.8
Total patients with GD	1432	100

The incidence of complications in relation to the type of operation is shown in Table 4. Postoperative hypoparathyroidism, was most frequent in patients who underwent TT, 31 (3.2%), whereas for patients who had D it was present in 8 (3.6%), and 3 (1.3%) for patients with STT.

**Table 4:** **Distribution of operated according to the type of operation for Graves’ disease and non associated and associated postoperative complications**

**Method**	**TT**		**D**		**STT**		
	n	%	n	%	n	%	sig
Postoperative permanent hypoparathyroidism	31	3.2	8	3.6	3	1.3	ns
Unilateral recurrent nerve paralysis	9	0.9	1	0.5	2	0.8	ns
Postoperative bleeding	10	1.0	1	0.5	2	0.8	ns
Postoperative permanent hypoparathyroidism with postoperative bleeding	5	0.5	2	0.9	1	0.4	ns
Unilateral recurrent nerve paralysis with postoperative bleeding	3	0.3	1	0.5	0	0.0	ns
Postoperative permanent hypoparathyroidism with unilateral recurrent nerve paralysis	2	0.2	0	0.0	0	0.0	ns
**Number of operated**	974	100	221	100	237	100	/

Postoperative unilateral recurrent nerve paralysis was reported in 9 (0.9%) patients who had TT, 1 (0.5%) who had D, and 2 (0.8%) who underwent STT. Bilateral vocal cord paralysis was not noted.

In patients who underwent TT postoperative bleeding occurred in 10 (1%), with D in 1 (0.5%) and in the group with STT in 2 (0.8%).

The incidence of complications was not statistically significantly different in relation to the type of surgical intervention (Fisher test, p > 0.05).

Frequency of associated complications is still lower than the frequency of non associated complications: permanent hypoparathyroidism with bleeding 5 (0.5%) in the TT group; 2 (0.9%) in the D group and 1 (0.4%) in the STT group. Post-operative unilateral recurrent nerve paralysis with bleeding was reported in 3 (0.3%) in TT group; 1 (0.5%) in the D group, and without associated complications in the STT group. Permanent hypoparathyroidism with postoperative unilateral recurrent nerve paralysis was present in 2 (0.2%) patients in the TT group and without these associated complications in the other groups (Table 4.).

The incidence of associated complications are also not statistically significantly different in relation to the type of surgery (Fisher test, p > 0.05).

By the end of the twentieth century, STT and D were the most commonly applied operation in the surgical treatment of GD on the grounds that the complications (postoperative hypoparathyroidism and recurrent laryngeal nerve palsy) were less frequent compared to TT. At the beginning of the twenty-first century, it became clear that STT and D were linked to a high risk of relapse of GD [9,10] and surgeons in specialized centres started to use TT as the operation of choice in the treatment of GD. In addition to that, the fact that TT removes the target organ for GD leads to the elimination of the autoimmune stimulus and improvement of the ophthalmopathy in these patients [11]. Also, TT permanently removes the risk of cancer in patients already having it, as TT represents an adequate surgical intervention both for GD and for carcinoma of the thyroid gland [9].

The series in our study represent uniform patient data obtained from a single institution and operated by four surgeons. According to available literature, our series are the largest single series of patients operated for Graves’ disease in a single institution. A larger number of patients was only shown in a study of Palit et al., but this study is a meta–analysis of 35 clinical studies and included 7241 patients [2].

Our study showed that TT is a safe and effective method of treating GD and has low complication rates.

Even when the initial treatment is surgery, the choice of the primary treatment option depends on the adequate cooperation of an endocrinologist, a specialist in nuclear medicine and an endocrine surgeon [12]. The choice of an adequate operation for GD is still the subject of debate [13], although after the year 2000, TT has become the treatment of choice for GD in many highly specialized centres in the world [9,14,15].

Of the total number (1432) of treated patients with GD significantly more were women 1211 (84.6%), while there were 221 men (15.4%), which roughly corresponds to the ratio 6 to 1. Analyzing our series of patients we did not find any significant differences in relation to gender in comparison to other authors [13,16]. The average age of the patients was 34.8 years (21 to 76) which is slightly higher than data available in literature [2,16].

In the US, the number of patients with GD that are surgically treated is small. According to the data of the American Thyroid Association, surgery is, in the US, a method of treatment in only 2% of patients with GD and only in 7% of patients with GD and thyromegaly [17].

Total or near total thyroidectomy is recommended for patients with ongoing thyroid cancer, those who refuse radio-ablation as a therapeutic procedure, or have a life threatening reaction to antithyroid drugs such as vasculitis, agranulocytosis and liver failure [9].

Total thyroidectomy is recommended to patients with Graves’ ophthalmopathy to eliminate the autoimmune stimulus from the orbital antigens [11,18].

Due to a relatively low rate of complications for TT, compared to less extensive procedures, many authors recommended TT as the treatment of choice for GD [16,19-21].

In our study the most common indication for surgery was thyromegaly (65%), followed by ophthalmopathy and ultrasound diagnosed thyroid nodule >1 cm (10%).

In the study of Geeta et al., in a series of 103 patients operated for GD, indications for surgery were 18% for thyromegaly, 26% for identified thyroid nodule and 20% for ophthalmopathy [14].

In our study, thyroid cancer was found, on definitive histopathological examination, in 146 patients (10.2%) with GD. Carcinomas smaller than 1 cm, or microcarcinomas, were present in 129 patients (9%), while greater than 1 cm were present in 17 patients (1.2%).

The incidence of thyroid cancer in GD by other authors is from 0% to 21% [22,23]. In our study it was 10.2%, but on a much larger number of patients than in the studies of the cited authors Gabriele et al. (64) [22] and Calo et al. (71) [23]. In the study by Geeta et al., thyroid cancer was found in about 8% of studied patients, in a series of 103 patients operated for GD [14].

In the study by Mittendorf and McHenry in a series of 32 patients with GD, carcinoma was found in 6% of operated patients [21], and the study of Pellegriti et al. found 4.7% of apparent clinically manifest carcinoma and 3.3% of occult thyroid carcinoma in a series of 450 patients with GD [24].

In our study, postoperative permanent hypoparathyroidism, was most frequent in patients who underwent TT (3.2%), whereas it was lowest (1.3%) among patients who underwent STT. Postoperative unilateral recurrent nerve paralysis was most common in patients who had TT (0.9%) and STT (0.8%), while it was much rarer in patients who had D (0.5%). Postoperative bleeding was more frequent in patients with TT (1%), than in patients with D (0.5%) or STT (0.8%).

In the study of Palit et al., (meta-analysis of 35 clinical studies with a total of 7241 patients) it was reported that there were no significant differences for any violation of the recurrent nerve (0.7% of STT compared to 0.9% for the TT) or for postoperative permanent hypoparathyroidism (1.0% for the STT compared to 0.9% for the TT) [2].

In the study of Wilhelm et al., in a series of 136 patients, they recorded hematoma in the neck in only one patient (0.8%) [10]; while the study of Jenkinks and Backer states that TT performed by an experienced surgeon has a rate of temporary recurrent nerve palsy of less than 1%, while postoperative bleeding ranges between 0.3% and 0.7% [25].

The incidence of complications in patients operated for GD in our series is low and is similar to the frequency of complications in other specialized centres in the world. About 65% of patients in the study of Mittendorf and McHenry have developed transient postoperative hypoparathyroidism [21] and the extent of surgical intervention alone is taken as an important influential factor of transient and permanent postoperative hypoparathyroidism [26].

The low incidence of complications in our study is the reason why we advocate TT in the surgical treatment of GD.

In relation to the type of surgery we did not find any significant differences in any of the two groups of complications.

The high incidence of postoperative permanent hypoparathyroidism, and associated complications (hypoparathyroidism with bleeding), in patients who underwent D is not a surprise. Although D operations are less radical than TT, in recent years, D is performed as a coerced surgical procedure for those patients in which we did not reliably identify the recurrent nerve and/or preserved the vascularisation of the parathyroid glands on the first side of the operated thyroid gland.

## Conclusion

If TT, as a surgical procedure performed in highly specialized institutions, is not accompanied by a higher rate of complications than less extensive surgery of the thyroid gland then this operation has the advantage over less extensive procedures for preventing relapse. There is no need for reoperation as it represents an adequate surgical treatment for thyroid cancer that can occur in patients with GD. If performed by an experienced surgeon, TT is the operation of choice for patients undergoing surgical treatment for GD.

## Abbreviations

D: Dunhill operation
GD: Graves’ disease
STT: Bilateral subtotal lobectomy
TT: Total thyroidectomy
